# Current and Emerging Advanced Techniques for Breeding Donkeys and Mules

**DOI:** 10.3390/ani15070990

**Published:** 2025-03-29

**Authors:** Andrés Gambini, Joanne M. Smith, Rhiannon J. Gurkin, Patricio D. Palacios

**Affiliations:** 1 School of Agriculture and Food Sustainability, The University of Queensland, Gatton, QLD 4343, Australia; r.gurkin@uq.edu.au (R.J.G.); p.palaciosbenitez@uq.edu.au (P.D.P.); 2 School of Veterinary Science, The University of Queensland, Gatton, QLD 4343, Australia; joanne.smith1@uq.edu.au

**Keywords:** sperm, oocyte, embryo transfer, ICSI, OPU, cloning

## Abstract

Donkeys and mules have historically played an important role in agriculture and are now gaining recognition for their contributions to animal conservation, milk production, tourism, and equid-assisted services. However, their distinctive reproductive challenges pose obstacles to breeding management. As a result, the application of assisted reproductive technologies (ARTs) could help address these challenges, enhancing their roles in both traditional and emerging industries. This review examines the current and emerging *in vitro* techniques for breeding donkeys and mules. Key methodologies such as sperm cryopreservation, innovative sperm preservation technologies, embryo transfer, *ovum* pick-up (OPU), oocyte maturation, and vitrification are discussed, emphasizing their importance in optimizing ARTs. Advances in *in vitro* embryo production technologies such as *in vitro* fertilization (IVF), intracytoplasmic sperm injection (ICSI), and somatic cell nuclear transfer (SCNT) are reviewed, with particular attention to its success in producing the first donkey and mule blastocysts or foals. Despite significant progress in the last decade, standardization of protocols for gamete conservation and embryo transfer are still required in long-ear equids. Advancing these technologies in combination with established *in vitro* embryo production could significantly improve reproductive outcomes and enhance the genetic management of donkey and mule populations.

## Simple Summary

Donkeys and mules have played a significant role in agriculture and are increasingly recognized as valuable species for conservation, milk production, tourism, and equid-assisted services. However, their unique reproductive challenges hinder breeding. This review explores recent advancements in reproductive technologies, including improved sperm preservation, oocyte collection, embryo transfer, and *in vitro* embryo production through fertilization and cloning. While challenges such as adapting horse protocols to this species and improving the preservation of reproductive cells remain, these innovations promise to optimize breeding processes, conserve endangered species, and protect genetic diversity. Advancing these techniques contributes to a sustainable future for these animals.

## 1. Introduction

Donkeys and mules have historically played a pivotal role in agriculture, transportation, and various societal functions. The overall advent of mechanization, modern farming techniques, and the proliferation of motorized vehicles in the 19th and 20th centuries led to a decline in the traditional roles of donkeys and mules. However, their utility in agriculture remains relevant in many low- and middle-income countries where donkeys and mules continue to play vital roles in agriculture and transportation [1]. In many developed regions, these animals transitioned from essential working partners to roles in leisure, conservation, traditional medicine, equid-assisted services, and tourism. Moreover, domestic donkeys have been receiving more attention due to their valuable milk production, although with still low production yield and sustainability [2,3,4]; their use to develop technologies for wild donkeys [5]; and, of course, mule production. Mules are renowned for their resilience and ability to thrive in harsh environments, making them invaluable for transportation, especially in military operations and mountainous expeditions.

Assisted reproductive technologies (ARTs) are defined as a suite of biotechnological interventions such as gamete preservation, artificial insemination (AI), embryo transfer (ET), and *in vitro* fertilization (IVF) that facilitate controlled and targeted breeding. In the last decade, ARTs have become a cornerstone in animal production and preservation, enabling more efficient genetic selection [6], improved herd quality [7], and enhanced conservation of endangered species [8]. In horses, ARTs have reshaped the landscape of reproduction and breeding, offering breeders new opportunities to enhance genetic diversity, improve reproductive efficiency, and elevate overall industry standards. Moreover, increasing the efficiency and developing new ARTs could contribute to minimizing physical risks and stress associated with frequent animal handling, thus promoting better welfare standards in breeding programs. Thus, the rapid expansion of domestic horse breeding began to influence other members of the genus *Equus*, particularly donkeys, to support genetic preservation, improve breeding lines, contribute to the growing demand for donkey milk, and enhance mule production.

Although some advanced ARTs are well established for horses, achieving comparable efficiency in donkeys remains challenging. Artificial insemination has been a pivotal technique in enhancing donkey and mule production by enabling more strategic breeding decisions, facilitating animal handling, and improving conception rates. Ongoing improvements in semen collection, semen processing, semen cooling, cryopreservation, and insemination protocols have further mitigated the constraints of natural mating, refining the genetic management of mule populations [9,10]. The protocols developed for domestic horses are not always directly transferable to donkeys due to species-specific differences. These variations have led to historically lower fertility outcomes following insemination in other equids, complicating the development of standardized freezing and thawing procedures. Furthermore, since mules are sterile hybrids, improving semen quality and AI protocols for donkeys is essential to sustaining and enhancing mule production. 

With the rapid development of *in vitro* horse embryo production, significant milestones have been achieved recently in other equids. The first domestic donkey [11] and mule [12] ICSI blastocysts (day 7–10 stage embryos) were reported, including an early embryonic vesicle of a donkey ICSI embryo transferred in a jenny recipient [13]. Interestingly, mules hold the distinction of being the first equids to be successfully cloned [14]. The genetic improvement of donkeys and mules, traditionally achieved through natural breeding and artificial insemination, could be significantly enhanced by the adoption of advanced *in vitro* embryo production technologies [5].

In this review, we will explore current and emerging advanced breeding technologies for donkeys and mules, including challenges with sperm preservation technologies, ET, oocyte collection, maturation, vitrification, and *in vitro* embryo technologies such as IVF, ICSI, and cloning. Table 1 provides an overview of key milestones achieved in the development and application of breeding techniques for donkeys and mules, highlighting progress in assisted reproduction and its practical implications for these species.

## 2. Challenges and Solutions in Donkey Sperm Preservation Technologies

The preservation of donkey sperm, encompassing both short-term chilling and long-term cryopreservation, remains a significant challenge due to unique species-specific characteristics and the limited availability of standardized protocols. Enhanced sperm preservation technologies are needed to reduce the need for repeated semen collection, thereby minimizing the stress and physical discomfort experienced by donor animals. Although donkey semen shares similarities with that of horses, notable differences in sperm cell morphometrics [24], cell components [4,12,25,26], seminal plasma constituents [26,27,28], and sperm kinematics, including velocity, trajectory, and motility patterns, [29] necessitate tailored preservation approaches [30,31]. Pregnancy rates after frozen–thawed insemination of donkey semen in jennies are generally lower compared to mares, highlighting not only the need to enhance sperm cryopreservation but also insemination protocols [32,33]. Approaches such as multiple deep horn inseminations with frozen–thawed semen [34], or resuspension after thawing in homologous seminal plasma [25], have been implemented to improve pregnancy rates in donkeys. A higher susceptibility of jennies to endometrial inflammation after insemination has been suggested as one of the major challenges for successful breeding outcomes in donkeys [35,36]. However, recent studies have observed no significant differences in endometrial responses in interspecific and intraspecific AI in domestic equids [37].

Cooled donkey semen often exhibits reduced AI outcomes compared to horse semen, prompting research into improved storage media and optimal cooling conditions [10]. Nevertheless, most practitioners have adopted stallion-based guidelines for preparing donkey semen for chilling and shipping. As with horses, sperm concentration and a proper extender are crucial requirements for optimum sperm refrigeration in donkeys [38]. Cooling donkey semen with commercial skimmed milk-based extenders does not consistently yield favorable outcomes, but fertility rates are better compared to egg yolk-based media [39]. Debates on practical adjustments, such as the need for seminal plasma removal, are still in place [4,36,40]. In this context, a recent study suggests that centrifugation is a fast, efficacious, and safe process compared to the filtration method [41]. More recent approaches, such as supplementation with cholesterol-loaded cyclodextrin in sodium caseinate-based or egg yolk-based extenders following seminal plasma removal, have shown promise in improving outcomes for chilling donkey semen [9,10].

Even though it has been over 60 years since the first report of freezing donkey semen [16], a universally accepted cryopreservation protocol for donkey semen remains elusive. Similarly to horses, large variability in the ability of sperm cells to withstand the freezing and thawing process between jacks has been reported [42]. Conventional freezing, which involves slow cooling rates, long equilibration periods, and permeable cryoprotectants (CPAs), remains the most common approach. While glycerol has been the predominant CPA for equine sperm cryopreservation, it has shown detrimental effects on donkey sperm [43]. Considering that outcomes without permeable CPAs are poor [44], dimethylformamide (DMF) could be the most suitable CPA for donkeys, with multiple studies supporting its efficacy [44,45]. Despite clear evidence of horse–donkey differences, a commercial extender, containing 1% glycerol and 4% methyl formamide, designed for horses is being used with acceptable outcomes in donkeys [46,47].

To enhance post-thaw sperm quality, freezing extender supplementation with molecules such as cholesterol-loaded cyclodextrin [48], L-proline [49], sucrose, and bovine serum albumin [44] are being tested. In parallel, optimization of thawing protocols by refining thawing temperatures and employing either rapid freezing or controlled thawing techniques continues to be explored as a means to further improve sperm functionality and viability [45,50]. More research in this area could help identify lipidomic [51] and proteomic profiles [28], providing a deeper understanding of molecular differences across species. This could lead to improved preservation protocols for donkey sperm.

Finally, beyond conventional freezing and chilling, experimental approaches such as sperm vitrification and ultra-rapid cooling to a glass-like solid state, which aim to avoid ice crystal formation [52], freeze-drying, removal of water from a frozen sample through sublimation to preserve it in a stable, dry form [53], and donkey sperm sexing, are under investigation [54]. However, these methods have yet to reliably demonstrate their capacity to produce healthy offspring. Sperm vitrification has the potential to achieve similar outcomes to conventional freezing [55], with the added benefit of faster recovery from uterine inflammation post-insemination. Optimization of donkey and horse sperm vitrification has recently been reviewed in detail [56]. No embryos have been reported after using donkey freeze-dried sperm to produce mules *in vitro* [53]. Accordingly, these novel sperm preservation technologies need further development in donkeys.

## 3. Embryo Transfer and Cryopreservation in Donkey and Mules

Embryo transfer (ET) involves flushing the donor’s uterus to collect embryos, typically seven to eight days post-ovulation, and transferring them into synchronized recipient females. ET is a valuable reproductive biotechnology widely used in multiple livestock species. In horses, the first successful ET attempts were reported in 1972 [57], and it is now a well-established reproductive biotechnology for many horse breeds. This technology presents numerous advantages for breeders and the industry, including the ability for high-performing or genetically superior female donors to produce multiple offspring within a single breeding season, circumventing the physical limitations of pregnancy on the donor, preserving both health and performance, while facilitating faster genetic improvement within the same bloodline. Despite being widely adopted in horses, the costs associated with donor and recipient management, embryo handling, and the availability of skilled personnel limit the application of ET [58]. In other equids, this is even more evident due to lower embryo recovery and transfer success rates [32,59,60] and the lower value of donkeys and mules compared with horses. Additionally, the limited number of recipient females for donkey or mule embryos presents further challenges [32].

After over a decade of suboptimal outcomes, the application of ET and cryopreservation techniques, such as embryo vitrification, in donkeys transitioned from horse-based protocols to methodologies specifically adapted to the donkey species. Although more research is needed, acceptable pregnancy rates (~45%) can be obtained when transferring fresh donkey embryos to donkey recipients using a non-phosphate-base embryo-holding media [61]. Moreover, embryo recovery rates on day 8 are significantly higher compared to other recovery days in donkeys [62], and on day 8, donkey embryos are more similar in size compared to horse or mule embryos [60,63,64].

Reports for short or long-term preservation of donkey embryos are limited but have been demonstrated as feasible techniques and were comprehensively reviewed [32]. Embryo survival after transfer is influenced by the diameter of embryos before vitrification, a trend that aligns with observations in horses [62,65]. The Cryotop method has emerged as a reliable approach for vitrifying donkey embryos [66]. Additionally, the removal of blastocoele fluid enhances success rates in donkeys [62] as reported in horses [67], together with the possibility of performing preimplantation genetic diagnosis, such as embryo sexing [68]. However, the technique for blastocoele fluid removal requires special considerations as improper handling may adversely affect outcomes [69]. Therefore, it is crucial to continue refining embryo cryopreservation protocols to expand and enhance the application of ET in donkeys.

ET technology has been essential for contributing to a better understanding of equine reproductive physiology [70], specifically interspecies ET within the *Equidae* family. In this area, the contribution of Dr. Professor William Richard Allen has been remarkable [71]. In his early studies, he demonstrated that equids possess a remarkable and rare ability to carry interspecies hybrid pregnancies to full term, as well as conceptuses generated via cross-species ET [19,20,72]. These studies revealed that horse embryos transferred into jennies experience a high rate of gestational failure compared to donkey embryos transferred into mares. Interestingly, the small number of mares capable of successfully carrying donkey fetuses to term often show consistent success in subsequent pregnancies following donkey embryo transfers. In contrast, mares that experience early pregnancy losses with donkey embryos are prone to recurrent pregnancy losses [73]. Additionally, donkey foals can be successfully produced using recipient mares when immunomodulation strategies [19] or hormonal treatments [61] are effectively implemented. The groundbreaking interspecies ET experiments vividly showcased the remarkable influence of the uterine environment on development. In a fascinating demonstration, mule demi-embryos, created by bisection, were transferred into both a jenny and a mare. This ingenious approach unveiled striking differences in placental development, driven by the interplay between genotype and the uterine environment [22].

Although rarely capable of natural conception [74], female mules can serve as recipients of equine embryos [20]. Both naturally and artificially cycling mules can successfully carry horse embryos to term, resulting in the birth of normal horse foals [20,75,76]. Mules have also been shown to carry donkey embryos to term [20,77,78]. Despite the long-standing use of ET technology in horses, mule-to-mule ET has only recently been reported [21]. In this case, three female mule foals were naturally delivered with no recorded placental abnormalities. Notably, recipient mules in late diestrus have also been shown to achieve pregnancy following the ET of a mule embryo [79]. These findings collectively highlight the potential of mules to serve as universal recipients for equid ET [76] which is particularly significant for the application of *in vitro*-produced embryos and conservation efforts.

## 4. Oocyte Collection, Maturation, and Vitrification in Donkeys and Mules

Oocyte collection, maturation, and preservation technologies represent critical advancements in ARTs for donkeys and mules, with significant future implications for both genetic improvement and conservation. Oocytes can be collected postmortem or via OPU from live animals. These techniques, combined with *in vitro* maturation protocols, have enabled the use of oocytes in advanced applications such as cloning and ICSI. Oocyte retrieval postmortem has been reported in domestic donkeys [11,80,81]. The procedure to recover these oocytes is, in essence, the same as described for mares [82]. In 2022, a comparative study examined the quantity, morphology, and maturation potential of cumulus–oocyte complexes (COCs) from abattoir-sourced ovaries and live jennies. The study revealed that COCs retrieved through OPU in donkeys exhibited superior quality compared to those obtained from abattoir-derived ovaries [81]. Similar findings are observed in horses where oocytes collected via OPU demonstrate enhanced developmental competence in applications such as cloning or ICSI, compared to postmortem retrieval [83]. Interestingly, mule and hinny germ cells within the ovary initiate meiosis after birth but the majority will degenerate. However, a small subset progresses through the early stages of meiosis and forms oocytes [84]. Thus, oocyte collection from mule ovaries is possible, and a study reported the recovery of mule oocytes from antral follicles postmortem [77]. Whether these oocytes can be successfully used for ARTs remains uninvestigated.

Oocyte collection through transvaginal ultrasound-guided follicle aspiration was first applied in domestic horses in 1992 [85]. After many years of refinement of the OPU procedure in mares, commercial equine OPU/ICSI programs are now well established worldwide. One of the primary factors influencing the success of the overall OPU program is the number of follicles aspirated from each donor mare. This number can vary significantly between individual animals [86], but a mean of 13.8 oocytes (range 1–48) could be recovered per mare, resulting in an average oocyte recovery rate of 53% [87]. The first published scientific attempt to retrieve donkey oocytes using OPU appeared in 2016 [23], although it is likely that earlier, unpublished efforts were made, especially considering that transvaginal aspiration was conducted in zebras in the 1990s [88]. Oocyte recovery rates through this technique in jennies range between 35 and 75% [11,13,23,81,89]. Preparing a jenny for an OPU session largely mirrors the approach used for mares, with detailed methodologies described in the previously cited literature. The primary considerations involve effective sedation and pain management [89,90]; addressing species-specific size and anatomical differences that can complicate ovarian manipulation, considering the risk reported for mares, such as peritonitis, rectal tears, hemorrhage, ovarian abscesses, and damage to the oviduct [87,91]; and implementing targeted strategies to closely monitor and minimize any potential welfare impacts on the animals.

The *in vitro* maturation conditions used for equine oocytes have been commonly adapted for donkey oocytes [11,13,89,92,93], obtaining between 50 and 70% of nuclear *in vitro* maturation rates. These IVM rates are similar to what is observed in mares. One of the most notable differences in donkey oocyte IVM is the longer time required for the extrusion of the first polar body, between 36 to 46 h [11,13,81] when compared to mares 24 to 30 h [94]. Whether this is due to suboptimal IVM conditions or a species-specific particularity remains to be studied. Limited research has been specifically focused on exploring more aspects of nuclear or cytoplasmatic oocyte maturation in donkeys. In this regard, specific genes such as IL10, CXCL10, and WGCNA may play a specific role in oocyte maturation in donkeys, distinct from other eutherian mammals [49,95]. Interestingly, blastocyst rates similar to horses, around 20 to 40%, can be achieved after ICSI in donkeys following horse *in vitro* culture protocols [13]. Although progress has been made in *in vitro* maturation in donkeys, the limited availability of jenny’s oocytes is slowing the advancement of *in vitro* breeding technologies compared to horses.

Finally, oocyte cryopreservation in donkeys is still in its infancy as no *in vitro* embryos have been produced from vitrified donkey or mule oocytes. The first attempts to vitrify immature donkey oocytes were only reported in 2019 [96], and while some oocytes resumed meiosis following *in vitro* maturation, their competence for fertilization and subsequent development were not evaluated. Oocyte vitrification remains a major area of development in domestic horses. Although foals have been produced from *in vitro* fertilization of vitrified oocytes [97,98], the impact on overall efficiency remains a significant challenge for large-scale commercial applications. It is likely that progress in this field will be made initially in domestic horses before being translated to donkeys.

## 5. *In Vitro* Embryo Production

*In vitro* embryo production encompasses several advanced reproductive technologies, including ICSI, IVF, and nuclear transfer technologies, including somatic cell nuclear transfer (SCNT) or cloning. These technologies offer significant potential for genetic improvement and are now integrated into programs in different livestock species, particularly cattle, but also in the horse breeding industry. *In vitro* embryo production is particularly promising for preserving endangered equids by enabling the production of multiple offspring from a single female or dead animal, increasing genetic diversity and reducing the need for extensive captive breeding programs.

Although some progress has been made in recent years in preserving the genetics of equids [5,99], as mentioned, the vitrification of in vivo-produced embryos in horses and donkeys remains challenging. Interestingly, equine embryos produced in the laboratory are smaller and have a less developed embryo capsule, which allows them to tolerate the process of vitrification better than their *in vivo* counterparts. Thus, embryo vitrification of in vitro-produced blastocysts presents a successful strategy for genetic conservation in the horse breeding industry. However, the application of *in vitro* embryo technologies in other domestic or wild equids has been limited due to restricted access to biological samples and funding constraints in this specialized field of research.

### 5.1. In Vitro Fertilization Techniques

IVF and ICSI are advanced reproductive techniques where fertilization occurs outside the female body within a laboratory setting, with IVF involving the co-incubation of oocytes and sperm and ICSI requiring the direct injection of a sperm cell into an oocyte; these methods have revolutionized human fertility treatments, enhanced animal production efficiency, and contributed significantly to the conservation of endangered species.

In donkeys, only one study attempted IVF without achieving blastocyst formation [23]. However, the recently established IVF protocol for horses described by [100], holds promise for improving this outcome. A key obstacle lies in determining if recently described horse sperm capacitation techniques can be adapted effectively for donkey sperm. Should this challenge be elucidated, an IVF protocol for donkey sperm could enable the production of donkey and mule embryos *in vitro* without requiring micromanipulation, making the approach more accessible. This advancement would also represent a significant step toward applying IVF technologies to conserve wild or endangered donkeys

Recent advancements over the past decade have made OPU and ICSI commercially viable in horses, revolutionizing the breeding industry [87,101]. These developments have enhanced ICSI efficiency beyond that of traditional assisted reproduction methods. Furthermore, recent progress in utilizing sexed semen in horse ICSI [102] has further driven this technology. However, as all the ARTs previously described in this review, the application of ICSI to other equids beyond horses has progressed slowly. In 2020, the first equid hybrid blastocysts were reported by injecting zebra sperm into horse eggs [103], and in 2023, the first domestic donkey blastocyst produced via ICSI was successful [11]. More recently, donkey blastocysts were successfully produced through IVM and ICSI, with the first transfer into recipient jennies, leading to an early vesicle stage, though it was not sustained [13].

In 2024, the first mule embryos produced via ICSI were documented [12]. This research described the localization of phospholipase C zeta in donkey sperm, a crucial molecule for oocyte activation, demonstrating differences from horses. Donkey sperm showed reduced activation efficiency, showcasing its functionality within closely related species. Moreover, no significant differences were observed in cleavage or blastocyst rates between mule and horse ICSI embryos, confirming that mule embryos can be generated *in vitro* at rates similar to those achieved in horses [12]. No pregnancies derived from *in vitro* ICSI embryos have yet been reported in mules.

Altogether, these findings suggest that horse-derived culture systems can effectively support donkey and mule ICSI embryo development *in vitro*. While further research is essential to achieve successful IVF blastocyst development in donkeys and mules, addressing the lower pregnancy rates observed after embryo transfer of both *in vivo* and *in vitro* embryos remains crucial. This is a fascinating area for further development as *in vitro* fertilization techniques facilitate the production of hybrids in the laboratory, contributing to a deeper understanding of the ability of members of the genus *Equus* to hybridize and successfully carry out interspecific gestation.

### 5.2. Cloning

Nearly three decades after Dolly’s birth, over 25 mammalian species have been cloned via somatic cell nuclear transfer (SCNT) [104]. Interestingly, the first equid cloned animal was a mule [14], followed by the first cloned horse in 2003 [105]. Since then, interest in equine cloning has grown [83,106,107]. Cloning is widely used for pets, agriculture, and sport horses, primarily in Argentina and the United States, by transferring a donor somatic cell into an enucleated oocyte. This approach bypasses the need for a sperm cell to produce an embryo, allowing for the propagation of specific genotypes and the preservation of elite, castrated, or deceased animals.

Cloning by SCNT is particularly interesting in infertile hybrids as it offers a pathway to overcome their reproductive barriers, conserve their genetic material, and explore their potential in research and conservation. The first mule clone was produced using fetal donor cells from a mule and *in vivo*-matured horse oocytes, following the oviductal transfer of over 300 early reconstructed embryos [14]. Nowadays, cloned horse foals are achieved by *in vitro* oocyte maturation and embryo growth to the blastocyst stage following conventional ET [106]. In the literature, at least four cloned mules have been reported [108,109], including Idaho Gem, Utah Pioneer, and Idaho Star, clones of a sibling of the champion mule Taz, born in 2003, and Lil Sarah, a clone of the champion mule Sarah Nelson, born in 2009. However, no scientific information is available on the health and viability of cloned mules.

Despite progress in cloning horses and mules, no successful donkey cloning has been reported. As this outcome is mainly due to the limited availability of donkey oocytes, advancing techniques for donkey OPU could make SCNT using donkey oocytes a viable option for preserving donkey genetics, including efforts to contribute to the conservation of the critically endangered Somali Wild Ass (*Equus africanus somalicus*). The use of cloning as a conservation tool for the *Equus* genus was initiated through the report of the first cloned foal of the endangered Przewalski’s horse (*Equus ferus przewalskii*) born in 2020 using a cell line preserved since 1980 [110]. However, this was achieved by using a domestic horse oocyte as a recipient cytoplast, known as interspecific SCNT. Similarly, zebra fibroblasts used as donor cells and horse oocytes as recipient cytoplasts successfully produced blastocysts, which are currently vitrified, with no apparent differences in embryo quality compared to cloned horse embryos [103]. Interspecific SCNT introduces mitochondrial DNA from the oocyte donor species, which may influence development, metabolic efficiency, and overall viability [111]. Selecting male clones for use in strategic breeding and conservation programs could help mitigate the transmission of undesirable mitochondrial DNA as it is typically not inherited through the male lineage (with some exceptions) [112]. Further research is needed to assess the long-term implications of interspecies SCNT and its relevance to conservation and breeding programs.

While cloning technology has the potential to enhance genetic variability in endangered donkey breeds and wild equid species by reintroducing lost genetic material if preserved samples are available, it also raises important ethical considerations and regulatory challenges [113]. A balanced approach is needed to weigh the feasibility of cloning for conservation efforts against its role in the commercial equine breeding industry.

## 6. Concluding Remarks

In conclusion, the advancements in ARTs for donkeys and mules, particularly in the last decade, have significantly expanded the possibilities for genetic improvement, conservation, and sustainable breeding of these equids. However, future efforts should focus on developing standardized artificial insemination and semen preservation protocols to enable the rapid expansion of the long-ear equid industry as these technologies are both cost-effective and easier to implement compared with more advanced ARTs. With *in vitro* embryo development in donkeys and mules now established, the next suggested steps involve refining recipient selection criteria and optimizing embryo transfer techniques. As these technologies continue to evolve, they hold great promise for securing the genetic preservation of donkeys and mules, bolstering conservation efforts, and meeting the growing demands for their diverse roles in agriculture, transport, and industry. This progress not only enriches our understanding of reproductive biology in these species but also reaffirms the importance of equids in global biodiversity and human livelihoods.

## Figures and Tables

**Table 1:** Pioneering key milestones in advanced breeding techniques for donkeys and mules.

Milestone	Year	Reference
Fresh Semen + AI—Donkey	1949 *	Mentioned in [15]
Frozen Semen—Donkey	1964	[16]
Frozen Semen + AI—Donkey	1975	[17]
Donkey into Donkey ET #	1997	[18]
Donkey into Horse ET (developed to term)	1982	[19]
Donkey into Mule ET (developed to term)	1982	[20]
Mule into Mule ET (developed to term)	2022	[21]
Mule into Donkey ET (pregnancy only)	1993	[22]
Mule into Horse ET (pregnancy only)	1993	[22]
OPU in Donkeys	2016	[23]
ICSI Donkey Blastocyst (donkey sperm into donkey egg)	2023	[11]
ICSI Mule Blastocyst (donkey sperm into horse egg)	2024	[12]
Clone Mule Foal (mule donor cell into horse ooplasm)	2003	[14]

AI, artificial insemination; ET, embryo transfer; OPU, *ovum* pick-up; ICSI, intracytoplasmic sperm injection; * approximate date; # unknown if developed to term.

## Data Availability

No new data were created or analyzed in this study. Data sharing is not applicable to this article.
